# Notes on Spanish Saffron

**Published:** 1867-06

**Authors:** Henry Biroth

**Affiliations:** Chicago


### NOTES ON SPANISH SAFFRON.

BY HENRY BIROTH, of Chicago.

Read before the Chicago College of Pharmacy.

From some remarks on saffron, at one of our meetings, I have been induced to test its value in regard to purity. I had three samples to dispose of, one from a New York importing house, the others from two Chicago wholesale houses. I was astonished to find them all extensively adulterated. In 100 parts of the saffron, I found

In sample No. 1, only 55 parts genuine crocus.
In sample No. 2, only 37 " "
In sample No. 3, only 42 " "

the rest were all colored flowers, mostly of marygold or calendula officinalis. It is no wonder that saffron is adulterated. It shares the fate of most of our costly imported drugs, such as opium, musk, the essential oils, etc.

The test is very simple and interesting. Put a few pieces of saffron on a glass plate, and touch them with concentrated sulphuric acid; the real stigmas assume a beautiful indigo-blue color, while the adulterations remain unchanged. Having experimented in this manner for a little while, you will get so well acquainted with the genuine saffron that you can readily separate it from the adulterations, with the pincers.

The U. S. Dispensatory, in speaking of the tinct. of aloes and myrrh, says:—The saffron, which has been retained in compliance with former prejudices, can add but little to the efficacy of the preparation, and being very expensive, has with great propriety been much reduced in the U. S. formula; and under the head of saffron, it says again:—*At present the chief use of the saffron is to impart flavor and color to officinal tinctures.* This is decisive to us. Now, allow me to ask you why the Pharmacopœa wishes to impose upon us a drug so costly and so much adulterated, merely for the purpose of flavoring and coloring three or four tinctures? Do these tinctures need a *coloring* substance, being too light without it, or is it necessary that they should be *flavored?* These tinctures are: Acetum opii, tincture of aloes and myrrh, and comp. tinct. of cinchona, which are sufficiently *colored* and sufficiently *aromatic* without saffron. We must seek some other reason. Saffron had been extensively used in olden times, and especially by the alchemists, who attributed properties to it which were nearer to superstition than to reality. The passion for gold, the mania of search for the philosopher's stone, the alcahest and the great elixir, or the red tincture, induced them to try almost everything; no wonder that saffron, whose tincture shows such a beautiful golden-yellow color, had to play also its part in this drama of development of Alchemistry.

Some of these old preparations, which contained saffron, are in use still in our Pharmacopœas, though greatly changed, of course. The first and most prominent of these is the great pharmaceutical and medical monstrum *theriaca*, now conf. opii, which will now celebrate its 2000th birthday, Mithridate, of Pontus, 88 A. Ch. being its inventor. It originally consisted of over 100 different drugs, and was always prepared in the City Hall with imposing ceremonies, under the protection of the municipal authorities; it was esteemed as a panacea for all diseases, internally and externally. Then the *sacred elixir*, now tr. of aloes and rhubarb, the *hiera picra*, the *elixir of paregoric* of Am. Disp. 1820, the *liniment. sapon. camph.*, according to Gray's Suppl. of Lond. Pharm., the *laudanum* of Lond. Ph.,

1720, the genuine formula of *Sydenham's tinct. of opium*, the *vinum rhei*, according to Gray's Suppl., the *tincture of rhubarb*, London, 1788. All these preparations orginally contained saffron, which in course of time was left out as the formulæ were changed more and more. Others retained the saffron as our:

*Tinct. of Aloes and Myrrh*, or the old elix proprietatis paracelsi.

*Pills of aloes and myrrh*, or Rufus Pills.

*Comp. tinct. of cinchona*, or Huxham's elixir.

*Acetum opii*, or the Quaker's black drops.

But even with these, it is only a matter of time. Such therapeutists as ALEXANDER, ORFILA, and MURRAY, have proved by experiments that crocus has but little activity, and the U. S. Dispensatory itself does not consider it a medicinal agent in its preparations, as before stated, merely using it for flavor and color.

To-day it is but a mistaken reverence for ancient formulæ—a fear of lifting the veil of mysticism. I do not intend to say that we shall not respect the Pharmaeopœa, or that we shall do as we please, or as we individually think more proper; on the contrary, the Pharmacopœa shall and must be our rule, our guide in pharmaceutical business, and we must adhere to it in our preparations, until a change is made. And here allow me to testify my high appreciation of the principles inculcated in the excellent essay of Mr. MILL, our Secretary, published in the Proceedings of Am. Ph. Ass., 1865, in which he recommends the utmost fidelity to our national Pharmacopœa. It is an essay full of ideas, which every young pharmacist, in particular, should observe and study.

But, on the other hand, the right of criticism, this solemn right, must not be denied to us, especially in regard to the Pharmacopœa. Each member should feel himself obliged to speak frankly about anything concerning the pharmaceutical business. *Ad astra per aspera.*

